# Photosynthetic Response of Blueberries Grown in Containers

**DOI:** 10.3390/plants12183272

**Published:** 2023-09-15

**Authors:** Melba R. Salazar-Gutiérrez, Kathy Lawrence, Elina D. Coneva, Bernardo Chaves-Córdoba

**Affiliations:** 1 Department of Horticulture, Auburn University, Auburn, AL 36849, USA; 2 Department of Entomology and Plant Pathology, Auburn University, Auburn, AL 36849, USA; 3 College of Agriculture, Auburn University, Auburn, AL 36849, USA

**Keywords:** photosynthesis, alternative production system, highbush blueberry, rabbiteye blueberry

## Abstract

Recently, there has been increased interest in container blueberry production as a viable alternative to open-field blueberry planting. Container production of blueberries offers numerous advantages, among these, a lack of limitation by suboptimal soil conditions in the open field and the ability to control substrate pH, drainage, and organic matter. The photosynthetic response for three container-grown Southern highbush blueberry (interspecific *Vaccinium* hybrids) cultivars including ‘Jewel’, ‘Meadowlark’, and ‘Victoria’ and a rabbiteye blueberry (*Vaccinium virgatum*) ‘Baldwin’, were measured during the spring and summer of 2022. It was hypothesized that the three cultivars evaluated would have different photosynthetic responses. The objective of this study was to determine the photosynthetic activity of different blueberry cultivars during the first year of crop establishment. A series of measurements were conducted every 2 h throughout the day and for different dates using a gas exchange data analyzer on newly matured fully expanded leaves located in the top middle section of the canopy for each cultivar. The response curves showed that net photosynthesis (A) became saturated at moderate light, with saturation occurring at a photosynthetic photon flux density (PPFD) of 1932 µmol m^−2^ s^−1^. At this point, the rate of CO_2_ assimilation was approximately 16.84 µmol CO_2_ m^−2^ s^−1^. No differences in (A) were found among cultivars. Overall, the attained values of photosynthesis provide a strong conceptual basis for understanding the cultivar variation response when grown in containers; therefore, the containerized system may serve as a production system for early fruiting blueberries in Alabama, USA.

## 1. Introduction

To meet the projected demand for food from limited agricultural land and rising populations, it is essential to enhance overall photosynthesis and thus productivity [1]. A recent report on crop prospects and the food situation released by the FAO indicated that countries are requiring external assistance for food. Prospects of persisting drought, due to rainfall shortages, and other extreme climate variability effects raise serious concerns about levels of acute food insecurity [2]. The next advance in field crop productivity will likely need to come from crop use efficiency of resources (e.g., light, water, and nitrogen), aspects of which are closely linked with overall crop photosynthetic efficiency [3]. Current trends in yield improvement of major field crops such as wheat, rice, maize, and soybeans are insufficient to meet the projected demand. Day by day, there is an increasing need to advance the knowledge of crops that could contribute to fulfilling the need for food worldwide.

Photosynthesis is the primary determinant of crop yield, and the efficiency by which a crop captures light and converts it into biomass over the growing season is a key determinant of final yield [4]. Plant growth and productivity rest ultimately on the photosynthetic activity of the crop. Plant growth models consider photosynthesis as the starting point for growth, resulting in biomass production accumulated over time. The maximum yield attainable from a crop has been termed yield potential and can be defined as the maximum yield attainable when the best-adapted crop variety is grown, in optimal conditions with no biotic or abiotic stress [5]. Determinants of yield potential are light availability, light capture, energy conversion, and plant architecture [6].

In addition to the demand for food production, the world must boost the output of food on existing agricultural land. Container/pot production offers many advantages, given that portability of the container allows farmers an option to deal with provisional land tenure, expanding the possibility of urban growing. Billions of acres of farmland are facing soil degradation. Containerized crop production can offer enhanced food production capabilities for communities through year-round production [7]. Due to specific requirements for low soil pH, new production methods are being introduced for highbush blueberries [8]. Container-grown crop producers can adopt research-based best management practices proactively. This allows them to minimize the economic and environmental risk of limited access to high-quality water or adapt production practices over time because of changing climate conditions [9]. One of the potential benefits of container production concerning cold protection is the ability to use much less water to achieve the same level of freeze protection.

Blueberry containerized production is a fairly recent approach [10], with an expanding trend in recent years [11,12,13]. Usually, in nurseries, blueberries have been propagated and grown as transplants in containers, but only for a short time before planting. It offers the advantage of not being limited by suboptimal soil conditions in the open field and the capability to control substrate pH, drainage, and organic matter [14]. Blueberries are a long-term perennial crop with soil pH requirements in the range of 4.5–5.5 [15], which is different than most other small fruit crops grown in containers for fruit production [16]. Container production in a limited space allows the moving of plants and adapting growing density based on plant growth [17]. Concerns for blueberry container production include a potential constraint on root growth for mature plants and the feasibility of long-term production [17]. There are various unknowns for containerized production of blueberries, and it is critical to understand the relationship between physiological responses to environmental factors and growth development, as well as determine how plants adapt to a changing climate. We aimed to determine the photosynthetic activity of selected blueberry cultivars during the first year of the crop establishment in containers, which allows an understanding of the photosynthetic responses of blueberry cultivars grown in containerized production systems.

## 2. Materials and Methods

### 2.1. Plant Material

**Figure 1:**
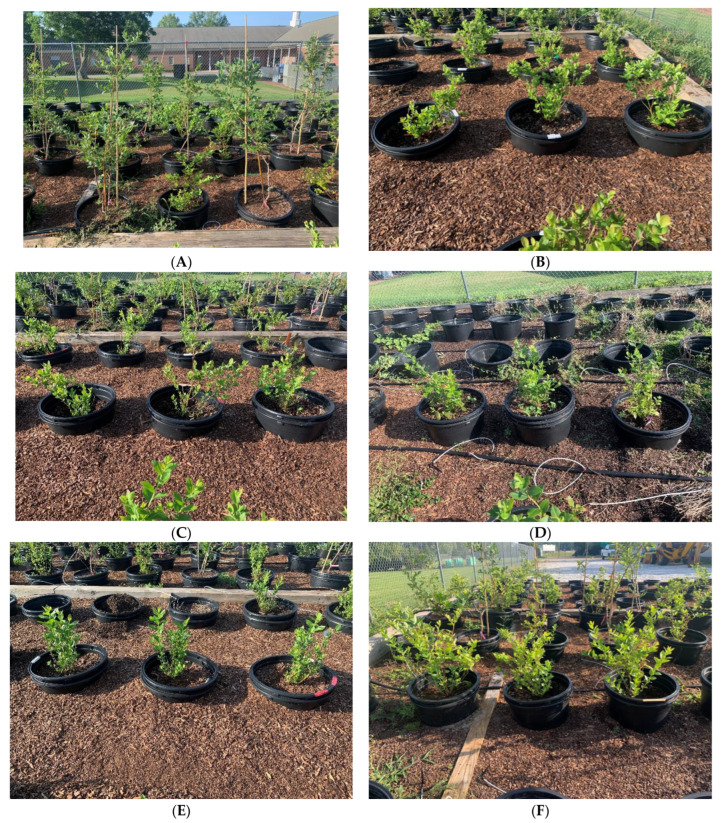
Cultivars utilized in the study, rabbiteye blueberry ‘Baldwin’ (**A**) and southern highbush blueberries ‘Jewel’(**B**), ‘Meadowlark’ (**C**) and ‘Meadowlark2′ (**D**), ‘Victoria (**E**) and ‘Victoria 2′ (**F**).

The experiment was conducted at the Plant Science Research Center ‘PSRC’ of Auburn University, located at (32°35′18″ N; 85°29′20″ W at 209 m Auburn, AL, USA). Four different cultivars of blueberries were considered in the study, including three Southern highbush blueberry species (Interspecific *Vaccinium* Hybrids) ‘Jewel’, ‘Meadowlark’, and ‘Victoria’ that came from Fall Creek Farm and Nursery, Inc. (Lowell, OR) and a rabbiteye blueberry (*Vaccinium ashei*) ‘Baldwin’ from Bottoms Nursery, LLC (Concord, GA) [18]. On 5 November 2021, a total of 90 plants distributed as 30 per cultivar were planted in pots for ‘Jewel’, ‘Meadowlark’, and ‘Victoria’. On 28 January 2022, 40 ‘Baldwin’ plants were planted in pots, and on 19 March 2022, a total of 120 plants (40 southern highbush per each of the cultivars ‘Jewel’, ‘Victoria 2′, and ‘Meadowlark 2′) were planted in pots (Figure 1).

**Figure 2:**
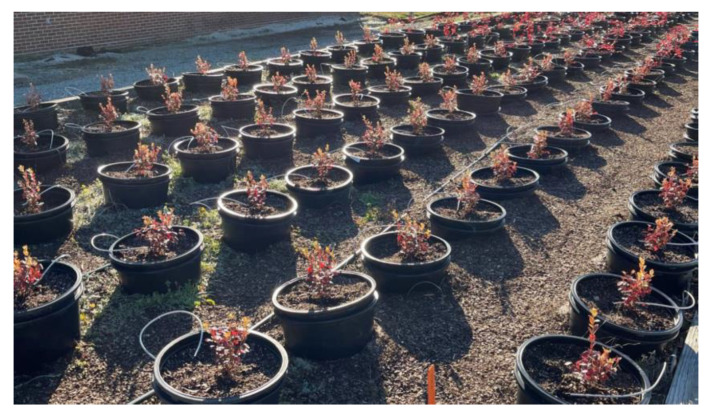
Experiment disposition in the pots and irrigation showing one emitter per pot.

Seven gallon plastic nursery pots were placed at a 0.91 × 0.60 m distance and then filled with a substrate of potting mix composed of 25% peat, 25% pine bark, and 50% ProMix Bx peat-based commercial growing media with perlite and vermiculite. Chemical analysis showed that the mix’s chemical characteristics are within the recommended range of pH for blueberry production at 4.79 [16], electric conductance value was 0.65, and soluble salts were 413 ppm. The irrigation method used was a low-pressure dripping system with one micro-emitter used per pot adjusted throughout the season (Figure 2).

### 2.2. Weather Conditions

**Figure 3:**
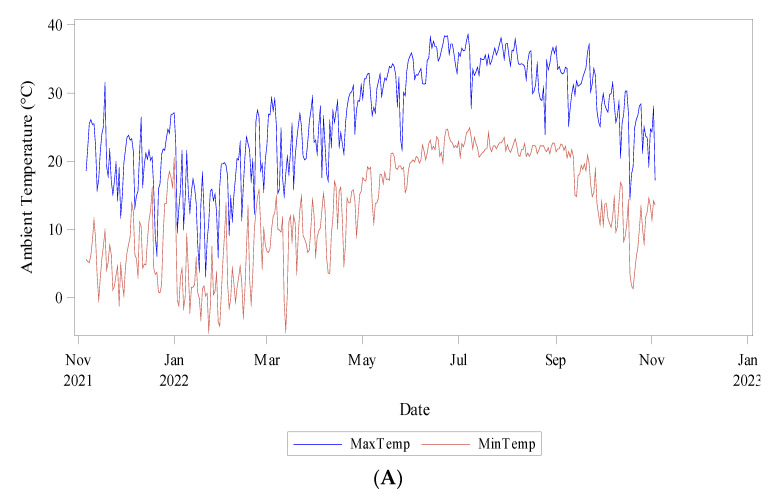

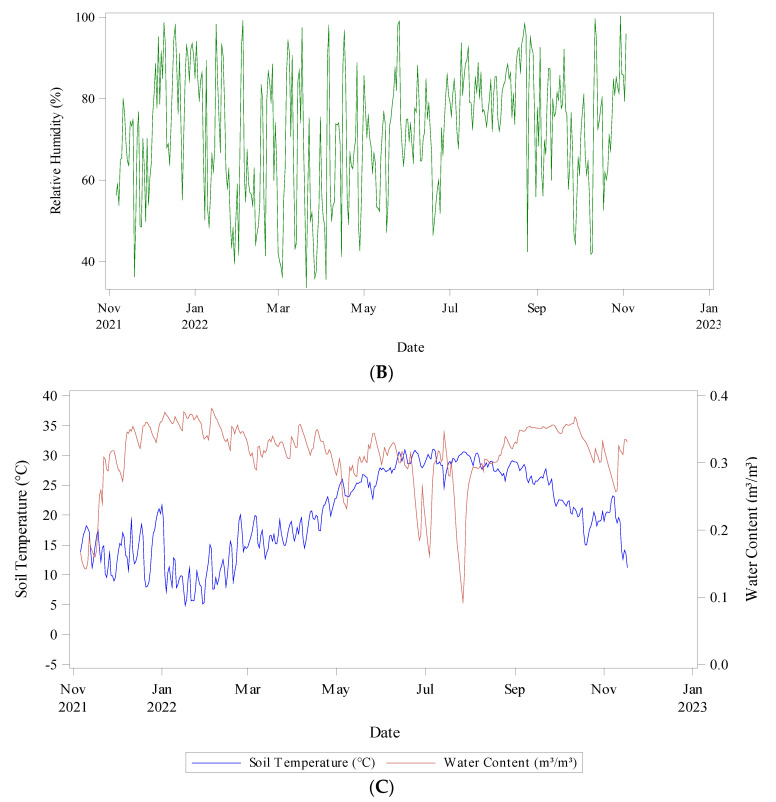
Climatic conditions during data collection (Nov 2021–Nov 2022) (**A**) Ambient Temperature (°C) minimum and maximum, (**B**) Relative humidity (%), and (**C**) Soil Temperature (°C) and Water Content (m^3^/m^3^).

Daily meteorological data was collected from six sensors located across the experimental plot. Weather variables and soil conditions were monitored constantly. A watchdog A150 sensor logging ambient temperature, relative humidity, and dew point temperature was used. Six Teros 11 meter sensors attached to a ZL6 meter logger recorded soil temperature and soil water content (Figure 3).

### 2.3. Gas Exchange Measurement

**Figure 4:**
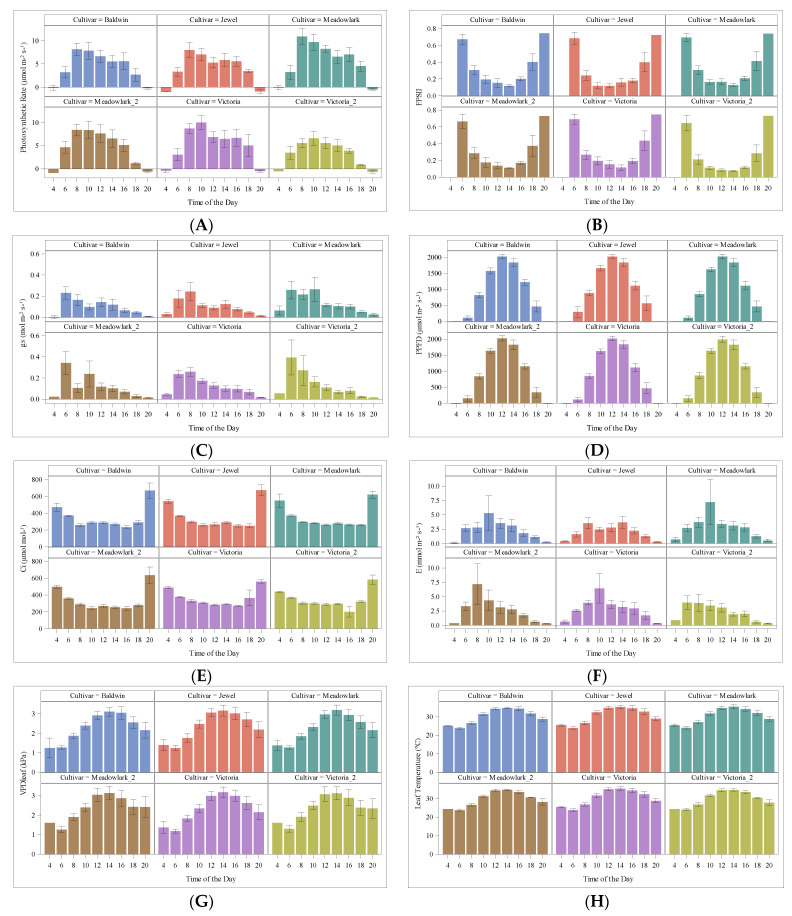
(**A**) A Daily photosynthetic rate, (**B**) F_PSII_ Photosystem II efficiency, (**C**) stomatal conductance (gs) values at saturated CO_2_, (**D**) Photosynthetic Photon Flux PPFD, (**E**) leaf intercellular CO_2_ concentration -intercellular carbon Ci, (**F**) transpiration rate E (mol m^−2^ s^−1^), (**G**) VPDleaf vapor pressure deficit at leaf temp (kPa), and (**H**) Air temperature (°C) (**H**) diurnal changes evaluated for all cultivars by time of the day measurement.

Gas exchange measurements were conducted using LI-6800 Portable Photosynthesis System (LI-COR Biosciences Inc., Lincoln, NE, USA) (Figure 4). Photosynthetic CO_2_ assimilation rate (A), stomatal conductance (g_s_), leaf intercellular CO_2_ concentration (Ci), transpiration (E), and Photosystem II efficiency (F_PSII_), photosynthetic photon flux (PPFD), vapor pressure deficit at leaf temp (VPDleaf), and Leaf temperature (Tleaf) for different blueberry cultivars were performed. Diurnal measurements were conducted every month from sunrise to sunset, starting in May and finalizing in August. From May to June, measurements were taken from 4:00 am to 8:00 pm, and for July and August, measurements were conducted from 8:00 am until 6:00 pm to observe the changes across the day. In addition, every two weeks a midday measurement was performed where the pick was identified in the diurnal measurements between 8:00 am to 3:00 pm every two hours.

On a newly matured fully expanded leaf located in the top middle section of the canopy, three to four independent replicate plants were used for each cultivar. The chamber calibrations were set to ambient conditions for CO_2_ approx. 410 µmol/mol to analyze the gas exchange response to the conditions of the potted plants. Current conditions of sunlight and temperature at the time of measurement and during the measurement dates were used for gas exchange parameters, and the current leaf temperature was set to the recorded ambient temperature. This changed by the time of the day and the month of the year, and PPFD values range between 1100–2300 µmol m^−2^ s^−1^.

### 2.4. Statistical Analysis

Descriptive statistics and a generalized linear mixed model were performed to evaluate the interaction among cultivars by the hour (hour during the day) with repeated measurements over time (month). According to the Akaike Information Criterion (AIC), the covariance matrix of the random effect (repeated measurements) was selected as the following Equation (1).

(1) $$AIC=2k-2\ln\left(\hat{L}\right)$$

where *k* is the number of estimated parameters in the model, and *L* is the maximum value of the likelihood function for the model. Fixed effects of cultivars were evaluated and compared using a Tukey–Kramer test with a significant level of *p* = 0.05. A Pearson linear correlation analysis was carried out for photosynthetic rate, stomatal conductance, intercellular carbon, quantum efficiency, light interception, air temperature, and transpiration. Principal component analysis was performed using PROC PRINQUAL to analyze the nonlinear relationships among the variables. All statistical analyses were done using SAS Software [19].

To do the ANOVA and comparisons, the autocorrelation structure of A was adjusted for the repeated measurements using a SAS macro and PROC GLIMMIX; out of 15 covariance matrices tested, the smallest AIC = 1079.19 found was the First Order Ante-dependence matrix.

## 3. Results

Diurnal Measurements

The photosynthetic CO_2_ assimilation rate (A) increased as the light irradiance increased; the highest A observed for the cultivars evaluated was reached between 8:00–10:00 am (Figure 4) at 24 °C–28 °C. ‘Baldwin’ 14.48 µmol m^−2^ s^−1^, ‘Jewel’ 15.05 µmol m^−2^ s^−1^, ‘Meadowlark’ 16.85 µmol m^−2^ s^−1^, ‘Meadowlark_2′ 14.70 µmol m^−2^ s^−1^, ‘Victoria’ 16.25 µmol m^−2^ s^−1^, and ‘Victoria_2′ 11.20 µmol m^−2^ s^−1^, respectively. Respiration values were obtained for early and late hours of measurements (4 am and 8 pm (20 h)) (Table 1).

There were no significant differences between cultivars and no interaction cultivar-time for A, g_s_, Ci, E, and F_PSII_. Only time (hours of measurements during the day) was significantly different (Table 2).

The least square means and Tukey–Kramer test comparison were performed, suggesting no differences in A among cultivars (Table 3).

The least square means and Tukey–Kramer test comparison was performed, indicating differences in time for A measurements and that the A changes depending on the hour of the day when the measurement was taken. Photosynthesis rates at 8 and 10 am were the highest, while 4 and 20 were the lowest and different from the others. LS-means with the same letter are not significantly different. The letters displayed do not reflect all significant comparisons. The following additional pairs are significantly different: (14,18), (16,18). (Table 4).

For all the variables including A, PPFD, gs, E, Ci, F_PSII_, Tleaf, and VPDleaf, significant differences were only presented with a time (hour during the day) *p* value < 0.0001, but no interaction was presented for any of the variables analyzed.

The Net CO_2_ assimilation rate (µmol m^−2^ s^−1^) for each of the blueberry cultivars evaluated shows similar behavior throughout the day. The highest values were recorded for the first planted mature cultivars of ‘Meadowlark’ and ‘Victoria’, followed by ‘Jewel’ and ‘Baldwin’; the late planted and youngest ‘Victoria’ presented the lowest values (Figure 4A). Other gas exchange parameters measured were F_PSII_, and effects on the light response were observed with the highest values early during the day and late in the evening. The F_PSII_ shows a decrease as time goes by during the day, with a minimum value observed at 14 h, rising again towards the end of the day; this behavior was consistent for all the cultivars (Figure 4B).

Stomatal conductance (gs) was measured at saturated CO_2_ (mol m^−2^ s^−1^). The values changed throughout the day and by cultivar according to temperature; thus, in order to avoid water loss, the higher the temperature, the lower the conductance (Figure 4C). Photosynthetic Photon Flux PPFD fluctuates during the day as the incident light intensity changes during the time of measurements (Figure 4D). Leaf internal CO_2_ concentration shows an inverse pattern related to A. Intercellular carbon Ci (µmol mol^−1^) decreases as light intensity increases throughout the day (Figure 4E). The transpiration rate response curve to irradiance for mature blueberry leaves € (mol m^−2^ s^−1^) varied among cultivars, showing an increase during the hours of higher activity, which was 8:00 am–10 am for Baldwin, Meadowlark, and Victoria, but 8:00 am to 14:00 for Jewel (Figure 4F). Diurnal changes of VPDleaf vapor pressure deficit at leaf temp (kPa) (Figure 4G) and the leaf temperature (°C) (Figure 4H) are presented.

A linear comparison matrix among variables shows several significant values, except for gs and PPFD, gs and F_PSII_, and E and Tleaf. In particular, A was positively correlated with PPFD, gs, E, Tleaf, and VDPleaf and negatively correlated with Ciand F_PSII_ (Table 5). To summarize, A possibly is affected by the other variables of the system, which at the same time are correlated among them.

**Figure 5:**
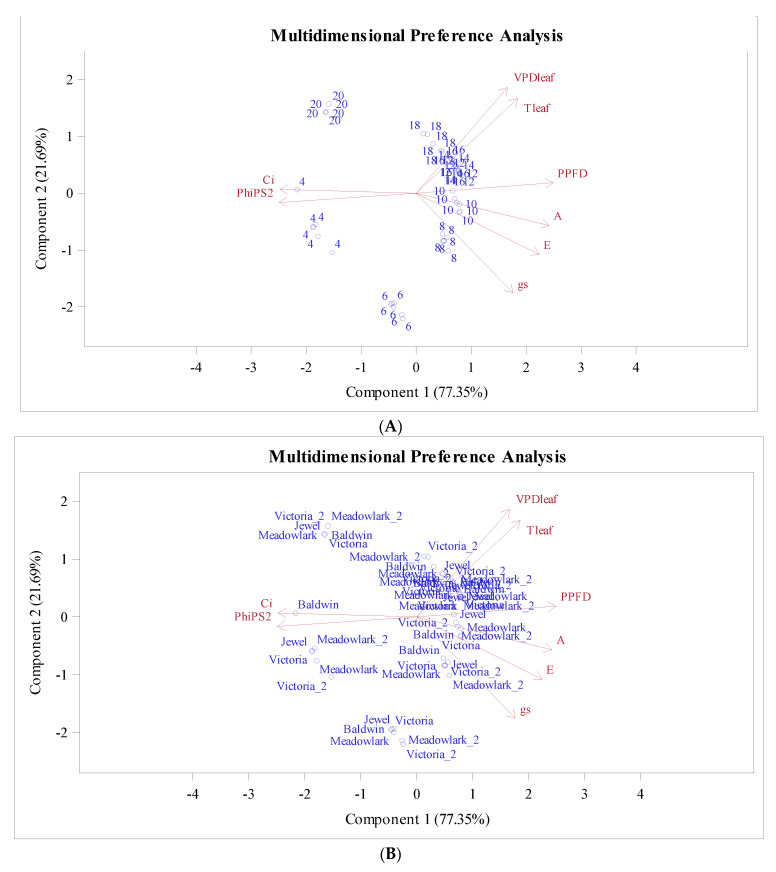
Transformed ratings projected into the two-dimensional plane of the analysis for hour (**A**) and cultivars (**B**).

To understand not only the linear correlation among measured variables but also nonlinear relationships, a monotone transformation principal component analysis PROC PRINQUAL in SAS was used to fit a principal component model with a nonlinear transformation of the variables. The PCA biplot (Figure 5) shows the hours (Figure 5A) and cultivars (Figure 5B) rating projected into the two-dimensional plane of analysis.

The variables that better fit the two-dimensional model are represented by the longer vectors, in this case, PPFD, F_PSII_, and Ci. Both components explain 99.04% of the total variance, component 1 (77.35%) and component 2 (21.69%), respectively.

Clusters are observed for hours as time goes on during the day (Figure 5A). Ci and F_PSII_ are highly correlated and associated with 4:00 am 6:00 am and 8:00 pm (20:00), while other hours tend to group in another cluster, with Ci and F_PSII_ close to the mean of each variable. PPFD is opposite to F_PSII_ and Ci, meaning that the efficiency and the intercellular carbon Ci decreases as PPFD increases and vice versa. Variables A, PPFD, E, Ci, and F_PSII_ explain the variation for the first component; hours from 8 to 18 had low values for F_PSII_ and Ci but high values for the rest of the variables (Figure 5A).

Regarding the cultivars, ‘Baldwin’ exposed a separation from the cluster at 4:00 am, when it is assumed that respiration is occurring at all times. For the rest of the day, all the cultivars are showing the same trend (Figure 5B). The VPDleaf and TLeaf are in the same quadrant close to each other, indicating a high relation. The corresponding correlation was positive and high (0.971) (Table 5).

## 4. Discussion

Some studies have been done on highbush blueberry production using containers, evaluating optimal pot size for greenhouse production [8,20] and estimating the impact on the substrate microclimatic conditions, metabolites content, and yield [8,21], while some others have focused on physical properties and substrate water distribution [22], as well as on analyzing the effects of rooting volume [23]. It is been suggested that reduced growth in smaller pots is caused mainly by a reduction in photosynthesis per unit leaf area, rather than by changes in leaf morphology or biomass allocation [23]. Photosynthesis of the blueberry cultivars used in this study grown in containers has not been studied before under Alabama conditions. In Mississippi, a study was conducted under high tunnels in containers to evaluate yield, timing of first berry harvest and peak harvest, single berry weight, and soluble solids content during the 2016 and 2017 growing seasons [10].

Previous work has reported that the blueberry crop presents high yield instability associated with the capacity to assimilate CO_2_ due to a year-to-year environmental variation [24]. Light and temperature are two of the most important environmental factors in crop production; the CO_2_ is assimilated in photosynthesis and its concentration increase in the atmosphere may influence carbon uptake and assimilation rate and, therefore, plant growth [25]. Photosynthesis increases as CO_2_ increases until it reaches saturating concentrations, as high CO_2_ allows plants to use the light more efficiently. The photosynthetic rate increases with temperature; however, the “optimum” temperature for photosynthesis depends on the CO_2_ concentration [26]. Excessive heat could reduce the efficiency of photosynthesis, impacting crop yields, the effect of which can impact the response of the other. Low temperatures can also impact the response of the photosynthetic rate. Temperature responses are also related to humidity, playing a role in stomatal conductance, affecting transpiration; the more the stomatal conductance, the more the capacity to gas interchange in the leaves [16,27,28]. Plants lose water in the process of CO_2_ absorption into the leaves, and it is a balance between photosynthesis, water loss, light absorption, leaf and air temperature.

Our initial analysis of photosynthetic responses of blueberries planted in pots under ambient conditions indicates that the rabbiteye ‘Baldwin’ and the three Southern highbush blueberry cultivars ‘Jewel’, ‘Meadowlark’, and ‘Victoria’ generate a light response under CO_2_ (410 µmol/mol), carbon assimilation rate obtained at light saturation intensities to a PPFD average ranged between 1035.8 to 1123.7 µmol m^−2^ s^−1^. These values denote notably higher values than those indicated in a highbush blueberries study conducted in Dundee, Scotland, UK, where photosynthesis was saturated at moderate light irradiance, and this was mainly due to stomatal and biochemical limitations. In a dynamic light environment, photosynthesis was further limited by a slow stomatal response to increasing light [24]. However, at high PPFD beyond 1000, µmol m^−2^ s^−1^ was slightly higher in the sun leaves than in the shade leaves of northern highbush blueberries [29].

On a clear day, PPFD shows a rapid increase in the morning, with a peak around midday and then a decrease rapidly afterward. A similar trend was found in a study done using different shade levels on a blueberry crop shade that significantly affected the growth of the ‘Bluecrop’ blueberry; with increasing shade level, shoot length increased, but the number of shoots per shrub decreased. Shade increased leaf and stoma size but decreased leaf thickness and stomatal density, being related to photosynthesis. Photosynthetic capacity was significantly depressed by shade. These acclimation responses of the ‘Bluecrop’ blueberry to shade affected reproductive growth characteristics and, ultimately, fruit yield [29]. Leaf internal CO_2_ concentration shows an inverse pattern concerning A, the intercellular carbon Ci (µmol mol^−1^) decreases as light intensity through the day increases; the same trend has been reported in previous studies [24]. Similar patterns but with a lower photosynthetic rate was found in three clones (‘Augusta’, ‘Brunswick’, and ‘Chignecto’) of lowbush blueberry (*Vaccinium angustifolium* Ait.) in a study conducted at the Agriculture and Agri-Food Canada Experimental Farm, Sheffield Mills, N.S. The photosynthetic assimilation rate for each clone was on average between 5 to 6 µmol m^−2^ s^−1^ for the vegetative year. Assimilation of CO_2_ increased with increasing photosynthetic photon flux (PPF) to between 500 and 600 µmol·s^–1^·m^–2^ in ‘Augusta’ and ‘Brunswick’, and to between 700 and 800 µmol·s^–1^·m^–2^ in ‘Chignecto’ [30], even though the values reported were lower. A similar trend was observed in our experiment in Alabama; the highest values for quantum efficiency were recorded during the first measurements of the day, even higher than the last measurement of the day. This indicates a limitation in the conversion of light energy to do photochemical work.

Other studies conducted at the Wild Blueberry Research Centre (WBRC) in Debert, Nova Scotia have determined the values of photosynthetic rate during the vegetative year as 4.0 to 5.0 µmol m^−2^ s^−1^ [31] and from 2.1 to 7.6 for wild blueberries, indicating that the wild blueberry is a plant with relatively low photosynthetic rates and is also a plant that is susceptible to photoinhibition [32]. Much lower yield in the wild blueberry system compared to cultivated highbush blueberries cannot be explained by low photosynthetic capacity; in contrast, the high maximum photosynthetic rates in wild blueberries suggest the potential to improve the yield of this unique agricultural system [33].

Temperature is another factor that could affect the photosynthetic rate. Temperature exerts a substantial influence on the growth, quality, and productivity of plants [34]. Low-temperature stress can reduce photosynthetic carbon assimilation capacity by inhibiting the stomatal conductance (gs) of blueberry leaves in a lowbush blueberry cultivar [35]. In our study, leaf temperature was positively correlated with photosynthetic rate (0.414) but negatively correlated with stomatal conductance (−0.287), which would indicate a reduction of photosynthesis by the inhibition of the stomatal conductance. Heat waves during the summer can cause containers to absorb and retain heat. High temperature in the soil can also affect photosynthetic rate and influence the growth and physiology of the blueberry plants.

The goal of this study was to determine the photosynthetic activity of different blueberry cultivars during the first year of the crop establishment, which allows an understanding of the photosynthetic responses of blueberry cultivars grown in containers. Highbush blueberry production in pots represents an alternative to soil production, where their pH, organic matter, and drainage demands can be maintained more easily using a selected substrate and controlled irrigation and fertilization [8].

It would be interesting to continue doing more measurements and perhaps focus on respiration in a future study to confirm that ‘Baldwin’, as a rabbiteye, has a different photosynthesis and respiration rate than the Southern highbush in a mature crop. This is just anticipation, since the photosynthetic and respiratory activity of fruit crops provide a crucial understanding of this primary determinant of crop yield and the efficiency by which a crop captures light and converts it into biomass over the growing season that needs to be confirmed with further studies. Another important piece would be to model and simulate fruit crop physiological responses under alternative production systems and different climatic conditions. Plant growth and productivity rests ultimately on the photosynthetic activity of the crop, and plant growth models consider photosynthesis as the starting point for growth, resulting in biomass production accumulated over time.

## 5. Conclusions

As this is a study to understand the photosynthesis of a recently established container blueberry production system, we can conclude that no significant differences in the photosynthetic rate by cultivar were found for the first year of the crop establishment, and no significant interaction between cultivar and time was found. However, significant differences were found during the time of the measurement, indicating that photosynthesis varies widely in response to light intensity and temperature over time during the vegetative stage of the crop.

## Figures and Tables

**Table 1:** Maximum daily photosynthetic CO_2_ assimilation rate of leaves A (µmol m^−2^ s^−1^) of four blueberry cultivars for measurements starting at 4 am and ending at 8 pm (20 h).

Cultivar	AM				PM				
	4	6	8	10	12	14	16	18	20
‘Baldwin’	−0.30	8.92	11.51	14.48	10.41	8.99	11.68	6.39	−0.18
‘Jewel’	−1.03	6.40	15.05	12.74	11.51	10.97	9.44	4.31	−0.34
‘Meadowlark’	−0.29	10.42	16.85	16.56	10.52	12.01	13.09	7.35	−0.34
‘Meadowlark_2′	−0.88	8.40	13.57	13.03	14.70	12.53	8.92	1.65	−0.38
‘Victoria’	−0.01	8.92	13.25	16.25	13.41	15.61	15.50	12.04	−0.26
‘Victoria_2′	−0.54	7.36	8.58	11.20	10.64	8.43	5.10	1.04	−0.40

**Table 2:** F-test for cultivar, time, and interaction.

Effect	F Value	*p*-Value
Cultivar	0.82	0.5406
Hour	18.38	<0.0001
Cultivar×Hour	0.52	0.9915

**Table 3:** Tukey–Kramer grouping for cultivar least squares means (Alpha = 0.05). LS-means with the same letter are not significantly different.

Cultivar	Estimate	
Meadowlark	6.0387	a
Victoria	5.5362	a
Meadowlark_2	5.4186	a
Baldwin	5.1511	a
Jewel	4.4781	a
Victoria_2	4.0246	a

**Table 4:** Tukey–Kramer grouping for hour (hour during the day) least square means (Alpha = 0.05). Means with the same letter indicate no significant differences.

Hour	Estimate	
10	8.7453	a
8	8.6977	a
12	7.1019	b
14	6.5579	bc
16	6.3970	bc
18	3.7961	c
6	3.5370	c
20	0.6260	d
4	0.5121	d

**Table 5:** Pearson correlation coefficients for physiological parameters considered.

	A	PPFD	gs	E	Ci	F_PSII_	Tleaf	VPDleaf
A	1	0.719	0.587	0.879	−0.771	−0.709	0.414	0.339
		<0.0001	<0.0001	<0.0001	<0.0001	<0.0001	0.0019	0.012
PPFD	0.719	1	0.135	0.630	−0.711	−0.893	0.810	0.751
	<0.0001		0.3282	<0.0001	<0.0001	<0.0001	<0.0001	<0.0001
gs	0.587	0.135	1	0.807	−0.333	−0.001	−0.287	−0.333
	<0.0001	0.3282		<0.0001	00.0138	0.9954	0.0356	0.0138
E	0.879	0.630	0.807	1	−0.609	−0.509	0.248	0.173
	<0.0001	<0.0001	<0.0001		<0.0001	0.0002	0.0698	0.2103
Ci	−0.771	−0.711	−0.333	−0.609	1	0.809	−0.540	−0.478
	<0.0001	<0.0001	0.0138	<0.0001		<0.0001	<0.0001	0.0003
F_PSII_	−0.709	−0.893	−0.001	−0.509	0.809	1	−0.716	−0.660
	<0.0001	<0.0001	0.9954	0.0002	<0.0001		<0.0001	<0.0001
Tleaf	0.414	0.810	−0.287	0.248	−0.540	−0.716	1	0.971
	0.0019	<0.0001	0.0356	0.0698	<0.0001	<0.0001		<0.0001
VPDleaf	0.339	0.751	−0.333	0.173	−0.478	−0.660	0.971	1
	0.012	<0.0001	0.0138	0.2103	0.0003	<0.0001	<0.0001	

## Data Availability

Data sharing not applicable.
